# The pulmonary blood volume measured by cardiovascular magnetic resonance imaging - relation to cardiac pumping and anthropometric measures

**DOI:** 10.1186/1532-429X-16-S1-P261

**Published:** 2014-01-16

**Authors:** Andreas Fredholm, Mikael Kanski, John Maret-Ouda, Magnus Lundin, Peder Sörensson, Håkan Arheden, Martin Ugander

**Affiliations:** 1 Department of Clinical Physiology, Karolinska Institutet and Karolinska University Hospital, Stockholm, Sweden; 2 Department of Clinical Physiology, Lund University and Lund University Hospital, Lund, Sweden

## Background

Non-invasive quantification of the pulmonary blood volume (PBV) by cardiovascular magnetic resonance imaging (CMR) is a novel method which may provide a measure of pulmonary congestion and backward failure in heart disease. The aims were to prospectively evaluate the factors contributing to PBV by CMR, and determine whether PBV is higher in patients with left ventricular (LV) measures outside normal limits.

## Methods

1.5T CMR was performed in 119 subjects (98 consecutive clinically referred patients, 21 controls). A subgroup of patients (n = 37, 38%) were identified by LV measures outside normal limits (Maceira AM, et al, JCMR, 2006; LV end-diastolic volume (LVEDV) index > 100 ml/m2, LV ejection fraction (LVEF) < 58%). PBV was measured as the product of the pulmonary transit time for an intravenous contrast bolus and cardiac output from CMR phase contrast flow measurement.

## Results

For all subjects, PBV correlated with height (R2 = 0.31, p < 0.001), LVEDV (R2 = 0.23, p < 0.001), lung volume (R2 = 0.20, p < 0.001) and age (R2 = 0.05, p = 0.02, multivariate adjusted R2 = 0.44, p < 0.001), but not with LVEF (R2 < 0.001, p = 0.91). Compared to controls (LVEDVi 86 ± 9 ml/m2, LVEF 61 ± 3%), the subgroup (LVEDVi 129 ± 31 ml/m2, LVEF 39 ± 11%) had a higher PBV (531 ± 133 vs 463 ± 91 ml, p = 0.04) but no difference in PBV indexed to lung volume (PBVI, p = 0.80), height (p = 0.41) or lung volume (p = 0.10).

## Conclusions

In a clinical population, only 44% of the variation in PBV could be explained by height, LVEDV, lung volume and age. Hence, quantification of PBV by CMR is a novel and feasible tool for clinical assessment of patients with heart disease and appears to provide additional information compared to conventional anthropometric and cardiac pumping measures. A subgroup of patients outside LV normal limits had a higher PBV but PBVI was not increased. Prospective evaluation of PBV in patients with congestive heart failure would be of value.

## Funding

Swedish Research Council, Swedish Heart and Lung Foundation, Stockholm County Council, Karolinska Institutet, Swedish Medical Assocation, Swedish Society of Medical Research, and The Kleberg, Osterman, Wiberg, Erling-Persson, and Grönberg Foundations.

